# Long-acting injectable antipsychotics linked to reduced short-term disruptive life event and death

**DOI:** 10.1371/journal.pmen.0000731

**Published:** 2026-11-10

**Authors:** Bing Han, Rebecca Butler, Karen J. Coleman, Rebecca C. Rossom, Xia Li, Heidi C. Waters, Claudia L. Nau

**Affiliations:** 1 Department of Research and Evaluation, Kaiser Permanente Southern California, Pasadena, California, United States of America; 2 Department of Health Systems Science, Kaiser Permanente Bernard J. Tyson School of Medicine, Pasadena, California, United States of America; 3 HealthPartners Institute Minneapolis, Bloomington, Minnesota, United States of America; 4 Global Value & Real World Evidence, Otsuka Pharmaceutical Development & Commercialization, Inc., Princeton, New Jersey, United States of America; PLOS: Public Library of Science, UNITED KINGDOM OF GREAT BRITAIN AND NORTHERN IRELAND

## Abstract

Long-acting injectable antipsychotics (LAI) may be beneficial for patients with schizophrenia (SCZ) or bipolar I disorder (BPI) compared with oral antipsychotics. We aimed to assess the association of LAI use on all-cause mortality and disruptive life events (DLE) among patients recently diagnosed with SCZ or BPI. We conducted a retrospective cohort study using adult patients meeting study eligibility criteria in Kaiser Permanente Southern California (KPSC) during 2007–2019. The index event for cohort entry was the first mental health related emergency department or inpatient encounter after initial diagnosis of SCZ or BPI. Two study arms were defined by the medication history within 180 days after index events: received LAI (LAI arm) and received oral antipsychotics only (oral-only arm). Unadjusted and adjusted two-sample comparisons were performed, where adjusted analyses used propensity score matching to balance observed characteristics. Outcomes were occurrence of specific DLEs (arrests, bankruptcy, and judgment filings), occurrence of any DLE, and all-cause mortality during the year after index events. The cohort had 3,667 patients in the oral-only arm and 218 in the LAI arm. Compared with the oral-only arm, the LAI arm had lower risks in all outcomes qualitatively. The rarity of the outcome and limited sample size in the LAI arm limited statistical power: adjusted risk difference was -1.68% for occurrences of any DLE (95% CI: -3.44%, 0.08%, p < 0.10), and -0.46% for all-cause mortality (95% CI: -0.98%, 0.06%, p < 0.10). Patients with SCZ or BPI taking LAIs may be associated with lower risk of DLE and mortality within the one-year period after index than those who took oral antipsychotics only. Larger studies are needed to further confirm these associations.

## Introduction

Schizophrenia (SCZ) and bipolar I disorder (BPI) are severe mental illnesses. Patients with diagnoses of SCZ or BPI often have recurring mental health related emergency room visits and hospital stays [1,2]. They also have an increased risk of experiencing disruptive life events (DLE) such as contact with the criminal legal system and financial difficulties [3–6] and increased risk of mortality and disability [7]. Antipsychotic medication is a key element of treating SCZ and BPI. Adherence to antipsychotic medications has shown to prevent relapse of psychotic episodes and re-hospitalizations and to reduce suicide and overall mortality [8,9].

However, adherence to oral antipsychotics has been historically and persistently low [10,11]. Low adherence rates have been attributed to the strong side effects of antipsychotics and other common challenges with medication adherence among patients with severe chronic illnesses, such as building a daily routine and managing complex treatment regiments [12,13]. In response to challenges with adherence, long-acting injectable antipsychotic medications (LAIs) have been developed [13]. Several studies have shown that LAIs are more effective than oral medications at improving adherence and reducing negative consequences of nonadherence [14–17]. While LAIs may provide some advantage over oral antipsychotic medications in terms of their stability of serum levels, overall side effects are likely comparable to other antipsychotic medications [18]. LAIs in the U.S. and Canada have traditionally been used as second line treatments for patients who have been struggling with relapse and non-adherence to oral treatments [13,19]. A relatively small study identified some unfavorable perceptions of LAIs, including concerns regarding their coercive image; however, these findings may not be generalizable to broader patient and provider populations [20].

A possible pathway linking LAI use to mortality and DLEs is through improved antipsychotic medication adherence. Improved adherence may reduce relapse and psychiatric destabilization and thereby potentially reduce the risk of downstream adverse outcomes. Some research suggests that use of LAIs may reduce the risk of suicide and all-cause mortality [21–23]. However, evidence is limited. Information on the effect of LAIs on DLEs, such as arrest and bankruptcy, is not available in the literature to our knowledge.

Our goal was to empirically investigate whether LAI use was associated with lower rates of DLEs and mortality during the 12 months following a first mental health crisis, defined as a mental health-related emergency department (ED) visit or hospitalization, among patients with SCZ or BPI. DLEs and mortality are complex outcomes influenced by many determinants beyond antipsychotic medication. This exploratory study did not intend to establish a causal mechanism but rather to provide empirical evidence that may inform future investigations of the potential mechanisms. Toward this goal, we used electronic health records (EHR) data from a large integrated health care system, Kaiser Permanente Southern California (KPSC), to identify patients with first diagnoses of SCZ or BPI at KPSC during 2007–2019. DLEs included arrest, judgement, and bankruptcy filing, which were retrieved from publicly available data warehoused by TransUnion [6]. All-cause mortality data were from KPSC’s EHR and the National Death Index.

## Methods

### Cohort of new patients with SCZ or BMI

KPSC is the largest health care provider in southern California with 4.8 million members in a 9-county area. KPSC’s members are approximately representative of the general population it serves in terms of demographic characteristics [24]. We identified diagnoses of SCZ using International Classification of Diseases, Ninth Revision, Clinical Modification [ICD-9-CM] codes 295.0 through 295.9, 297.1, 297.3, 298.8, or 298.9; International Statistical Classification of Diseases, Tenth Revision, Clinical Modification [ICD-10-CM] codes F20.x, F22-25, F28-F29. We identified diagnoses of BPI by ICD-9-CM codes 296.0x, 296.1x, 296.4x, 296.5x, 296.6x, 296.7x, 296.8x, excluding 296.82, and ICD-10-CM codes F30.x, F31.x, excluding F31.81. We required two outpatient diagnoses or one inpatient diagnosis to establish a qualifying diagnosis of SCZ or BPI. A newly diagnosed case was defined as a patient who had at least 12 months of KPSC membership (allowing for a maximum gap of 30 days in insurance coverage) prior to their first qualifying diagnosis between 2007 and 2019. To address potential confounding by disease severity and prognosis, we identified newly diagnosed cases at their first mental health related ED or inpatient encounter. Mental health related health care encounter included mental health diagnosis and other diagnoses that may be mental health related such as vertigo or dehydration. The index event was defined as the first mental health related inpatient or ED encounter. The index event and the first qualifying diagnosis could be during the same health care encounter.

Eligibility criteria included being between 18 and 65 years old, inclusive, at the time of index event (Fig 1). Patients who did not have a minimum of one-year follow-up period after the index event, those that had an LAI before the index event (i.e., were not LAI naïve), and those that did not receive any form of medication during the 180-day window after the index event were excluded.

**Fig 1:**
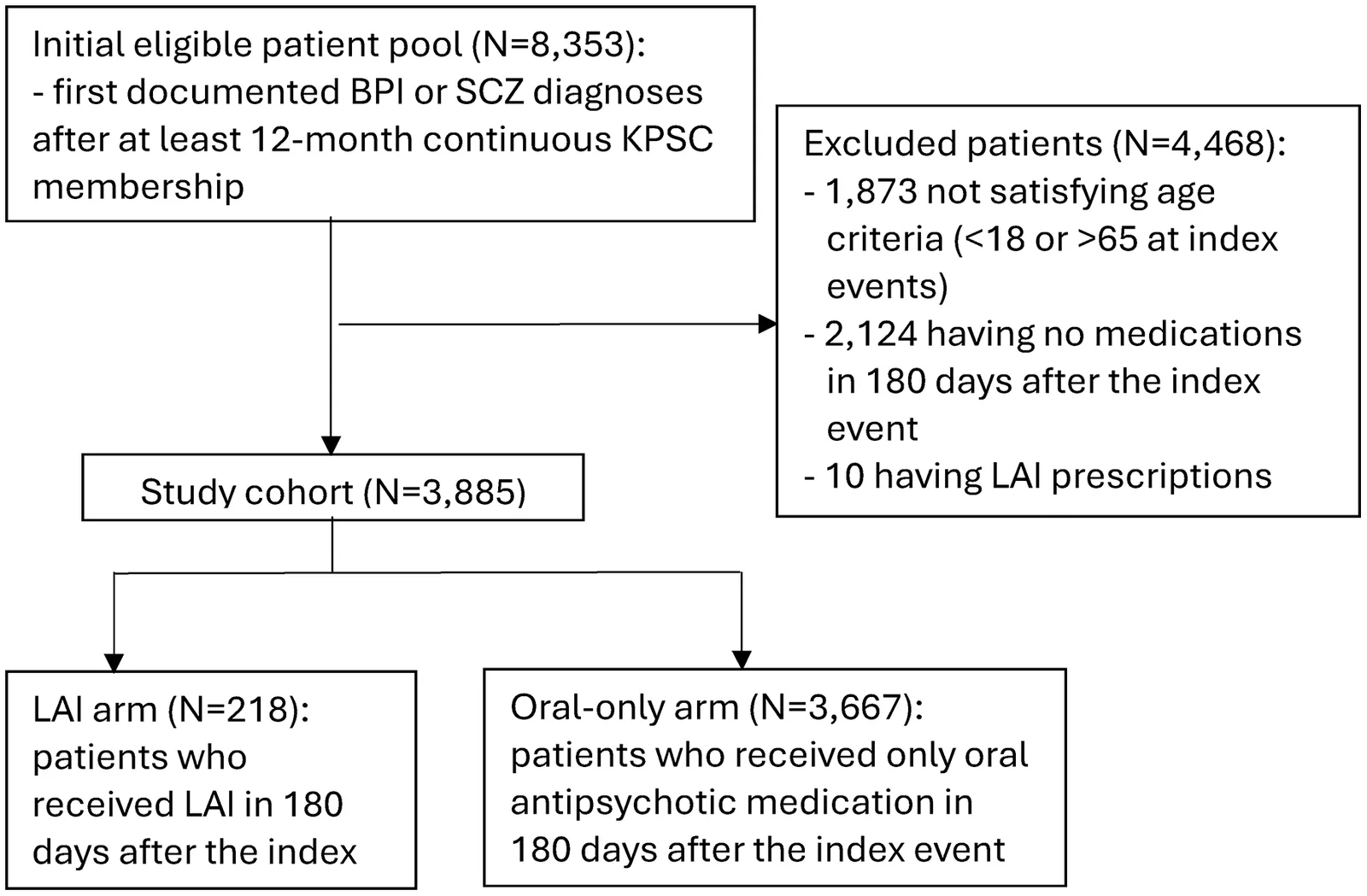
CONSORT diagram for the study cohort.

### Exposure and study arms

In this empirical study, *t*he exposure of interest was having at least one LAI administered within the 180-day window after the index event, regardless of concurrent oral antipsychotic use. The comparison group consisted of patients who received oral antipsychotic treatment but no LAI during this period. Thus, the exposure comparison was between LAI receipt, with or without concurrent oral treatment, and oral treatment without LAI receipt. We chose a relatively long window of 180 days to define the LAI exposure, since LAIs often require stabilization on the oral form of the medicine prior to the LAI administration or may not be the first choice of medication following an emergency encounter. The 180-day exposure period also yielded a slightly larger sample size for statistical analysis. The study cohort was divided into two mutually exclusive arms based on the antipsychotic medication history during the exposure period. The oral-only arm consisted of 3,667 eligible patients who received only oral antipsychotic medications and did not receive any LAI during the exposure period. The LAI arm received an LAI during the exposure period. Since LAIs were rarely prescribed as first line treatment, patients in the LAI arm often also received oral antipsychotic medications during the exposure period.

### Study outcomes and covariates

Data on all-cause mortality came from KPSC’s EHR and the national death index. Patients’ demographic characteristics, health care encounter history, and medication history were also from KPSC’s EHR. Data on DLEs including arrests, judgement, and bankruptcy filings were from TransUnion and have been described prior [6]. Records of DLE were publicly available court and police records and were assembled and warehoused by TransUnion, which did not include credit reports, credit scores, or other nonpublic financial information. More than 98% of eligible patients were linked to the TransUnion data. A multistep data-transfer process separated identifying information from clinical information, and the linked analytic data were de-identified and maintained on secure, firewalled, and encrypted servers. Linkages between the public data and EHR at the patient level were described in details in a prior study [6].

Since DLEs were rare, we defined three dichotomous outcomes as whether a specific type of DLE (arrest, bankruptcy, and judgement filing) ever occurred during the one-year period after the index event. We also defined a composite outcome of any of the three types of DLE occurrence during the 1-year study period. All-cause mortality instance during the study period was defined as the fourth binary outcome.

### Statistical analysis

We calculated descriptive statistics for the full study cohort and by study arms. Due to the rarity of all outcomes, we could not use logistic regression models for our analysis. Instead, we used two-sample comparisons of the risk of each study outcome. First, we conducted unadjusted two-sample comparisons between the LAI and the oral-only study arms. For each study outcome, we estimated the risk difference (RD) and the relative risk (RR). RD assessed the observed difference in risk of experiencing an outcome between the LAI arm and the oral-only arm as percentage points, where a negative value indicated a reduction of risk and a positive value indicated an increase in risk associated with LAI. RR calculated the ratio of risk between the LAI arm and the oral-only arm, where a value smaller than one indicated a reduced risk of experiencing the outcome of interest associated with LAIs and a value greater than one indicated an increased risk of experiencing the outcome of interest associated with LAIs. Next, we conducted adjusted analyses using propensity score matching, which was a semiparametric covariate adjustment technique [25,26]. We used a 3-to-1 matching ratio between oral-only and LAI patients. Matching dimensions included sex, race/ethnicity, age at index events, and primary diagnosis (SCZ vs. BPI). The calendar year of index events was also included in the propensity score to adjust for potential secular trends or unobserved time-varying effects potentially confounded with the outcomes [27]. The propensity score matching procedure was conducted using *library(MatchIt)* in R 4.3.1 without replacement. Covariate balances in the matched sample were assessed using standardized mean differences (SMD). Sensitivity analysis was performed to control for the 3-to-1 matching clusters in RD estimates. Wherever applicable, we applied the Farring-Manning score method to slightly increase statistical efficiency than the standard Wald’s z-test [28,29].

Because this study was exploratory, we reported p-values below 0.10 in addition to the conventional threshold of 0.05 and 0.01 [30,31]. Results with 0.05 < p-value<0.10 were not interpreted as statistically significant but were considered as weak signals for future hypothesis generation. Interpretation of these results focused primarily on the magnitude and precision of the estimates, as reflected by the point estimates and 95% confidence intervals.

All unadjusted and adjusted analyses were conducted in SAS 9.4. Transunion and KPSC EHR data retrieval started on 01/11/2020 (November 1st, 2020) and finalized (person-level linking and validation) on 11/04/2023 (April 11, 2023).

## Results

### Participants

Table 1 presents the descriptive statistics of the study cohort. Most patients were in the oral-only arm (n = 3,667; 94.4%). In the LAI arm (n = 218), most patients (89.0%) also received oral antipsychotic medications during the exposure period, i.e., the 180-day window after the index event. The majority of study patients were male (62.4%), with the LAI arm having a higher proportion of males than the oral -only arm (76.6% vs. 61.6%). The patient cohort was young, around 21.7 years old (SD = 3.2) at the initial qualifying diagnosis and 22.1 years old (SD = 3.3) at the index event with little to no difference between the two study arms. The study sample was racially and ethnically diverse. The majority of oral-only arm was Latino (39.1%) or white (34.1%), while a smaller proportion were African American (17.1%) or identified with another race or ethnicity (9.8%). Patients in the LAI arm predominantly identified as Latino (32.6%) or white (30.3%) and had notably higher representation of African Americans (25.2%) and patients who identified with another race or ethnicity (11.9%) than the oral-only arm. There were slightly more patients with SCZ than patients with BPI in the oral-only arm (52.5% vs. 47.5%). By contrast, most patients in the LAI arm had SCZ (77.5% vs. 22.5%). Overall, about 8% patients in both arms were insured by Medicaid. During the 180-day exposure period, patients were covered by oral antipsychotic medications for an average of 103.6 days (SD = 59.0) with little difference between the two arms. Patients in the LAI arm received an average of 59.2 days or LAI coverage (SD = 46.2).

**Table 1:** Study sample descriptives statistics and balance assessment between study arms. The “oral-only” and “LAI” columns are for the two study arms, respectively. The “matched oral-only” column is the subsample of the oral-only arm matched to the LAI patients by 3:1 propensity score matching. The two “SMD” columns report the standardize mean differences before and after matching.

Characteristics ^a^	Overall N = 3,885	Oral-only N = 3,667	LAI N = 218	Matched oral-only N = 654	SMD ^b^ LAI vs. oral-only	SMD ^b^ LAI vs. matched oral-only
Gender						
Male	2,425 (62.4)	2,258 (61.6)	167 (76.6)	507 (77.5)	0.36	0.02
Female	1,460 (37.6)	1,409 (38.4)	51 (23.4)	147 (22.5)	-0.36	-0.02
Age at initial diagnosis ^c^	21.7 (3.2)	21.7 (3.2)	21.3 (3.0)	21.2 (2.8)	-0.08	0.04
Age at index event	22.1 (3.3)	22.1 (3.3)	21.7 (2.9)	21.6 (2.7)	-0.16	0.03
Race/ethnicity						
Hispanic	1,503 (38.7)	1,432 (39.1)	71 (32.6)	223 (34.1)	-0.14	-0.03
White	1,315 (33.8)	1,249 (34.1)	66 (30.3)	187 (28.6)	-0.08	0.04
Black	682 (17.6)	627 (17.1)	55 (25.2)	172 (26.3)	0.19	-0.02
Other	385 (9.9)	359 (9.8)	26 (11.9)	72 (11.0)	0.07	0.03
Primary diagnosis						
SCZ	2,093 (53.9)	1,924 (52.5)	169 (77.5)	501 (76.6)	-0.60	0.02
BPI	1,792 (46.1)	1,743 (47.5)	49 (22.5)	153 (23.4)	0.60	-0.02
Year of index event	2014 (3)	2014 (3)	2014 (3)	2014 (3)	0	0
Had oral antipsychotics in 180 days after index events^c^	3,861 (99.4)	3,667 (100)	194 (89.0)	654 (100)	-0.35	-0.35
Medicaid ^c^	320 (8.2)	302 (8.2)	18 (8.3)	51 (7.8)	0.00	0.02
# Days under antipsychotic medications during the 180-day exposure window^c^						
Oral	103.6 (59.0)	103.4 (58.9)	106.9 (61.4)	103.2 (58.6)	0.06	0.06
LAI			59.2 (46.2)			

^a^n(%) for categorical variables and mean (SD) for numerical variables.

^b^Standardized mean differences.

^c^Not a matching dimension.

Table 1 also reported the characteristics of the subsample after the 3-to-1 propensity score matching procedure. Covariate balance on sex, race/ethnicity, and primary diagnosis was substantially improved after matching. Absolute SMDs for all balancing covariates were below 0.10 after matching.

### Disruptive life events and mortality

Table 2 presents the results of unadjusted two-sample analysis between the LAI and oral-only arms prior to propensity score matching. The prevalence rates of all study outcomes were lower in the LAI arm compared to the oral-only arm, with only 2 total outcomes of interest for the LAI arm (one arrest and one judgement filing). Prior to matching, compared to the oral-only arm, the LAI arm had significantly lower risks for all-cause mortality (RD = -0.65%, 95% CI: -0.92% to -0.39%, p < 0.001) and bankruptcy (RD = -0.60%, 95% CI: -0.85% to -0.35%, p < 0.001). A weak signal was also observed for a lower risk of any DLE in the LAI arm (RD = -1.29%, 95% CI: -2.64% to 0.06%, p < 0.10). RR estimates for all outcomes were less than one, indicating reduced risk associated with LAIs but none of the RRs was statistically significant.

**Table 2:** Unadjusted analyses for binary study outcomes during 1-year follow-up since the index event between the LAI and oral-only arms.

Outcomes	Oral-only (N = 3,667) n(%)	LAI (N = 218) n(%)	RD (%) ^a^ (95% CI)	RR^a^ (95% CI)
Simple outcome				
All-cause death	24 (0.65)	0 (0)	-0.65 (-0.92, -0.39)***	0 (0, 2.65)
Arrest	32 (0.87)	1 (0.46)	-0.41 (-1.36, 0.53)	0.52 (0.09, 3.00)
Bankruptcy	22 (0.60)	0 (0)	-0.60 (-0.85, -0.35)***	0 (0, 2.89)
Judgment	30 (0.82)	1 (0.46)	-0.36 (-1.30, 0.58)	0.56 (0.10, 3.20)
Composite outcome				
Any DLE	81 (2.21)	2 (0.92)	-1.29 (-2.64, 0.06) ^&^	0.42 (0.11, 1.51)

^a^RD is risk difference between LAI and oral-only arms. RR is relative risk between LAI and oral-only arms. Statistical significance level is marked as p-value < 0.001 ***, <0.01 **, <0.05 *, <0.10 ^&^.

Table 3 presents the adjusted analysis between the LAI arm and the matched oral-only arm (n = 654 after matching). Point estimates of RD and RR favored the LAI arm across all outcomes. However, statistical precision was generally weak due to the low number of outcomes of interest and the reduced sample size (Table 3). No estimate was statistically significant by the conventional p < 0.05 level. All-cause mortality had negative RD (adjusted estimate = -0.46%, 95% CI: -0.98% to 0.06%, p < 0.10). Bankruptcy had negative RD (estimate = -0.46%, 95% CI: -0.98% to -0.06%, p < 0.10). The composite outcome of any DLE also had negative RD (estimate = -1.68%, 95% CI: -3.44% to 0.08%, p < 0.10). Sensitivity analyses that further adjusted matching clusters gave very similar RD estimates and were hence omitted. All RR estimates were smaller than one, but all p-values exceeded 0.10.

**Table 3:** Adjusted analyses for binary DLE outcomes during 1-year follow-up since the index event between LAI and matched oral-only patients.

Outcomes	Matched (N = 654), n(%)	LAI (N = 218), n(%)	RD (%) ^a^ (95% CI)	RR^a^ (95% CI)
Simple outcome				
All-cause death	3 (0.46)	0 (0)	-0.46 (-0.98,0.06)^&^	0 (0, 3.82)
Arrest	6 (0.92)	1 (0.46)	-0.46 (-1.62,0.70)	0.50 (0.08, 3.13)
Bankruptcy	3 (0.46)	0 (0)	-0.46 (-0.98,0.06)^&^	0 (0, 3.82)
Judgment	8 (1.22)	1 (0.46)	-0.76 (-2.00,0.47)	0.38 (0.06, 2.28)
Composite outcome				
Any DLE	17 (2.60)	2 (0.92)	-1.68 (-3.44,0.08)^&^	0.35 (0.09, 1.35)

^a^RD is risk difference between LAI and oral-only arms. RR is relative risk between LAI and oral-only arms. Statistical significance level is marked as p-value < 0.001 ***, <0.01 **, <0.05 *, <0.10 ^&^.

## Discussion

This retrospective cohort study linked KPSC’s EHR data to credit reporting agency data to compare the risk of DLE and mortality outcomes between patients who received LAIs or oral antipsychotics only after their first mental health related ED or inpatient visit (index event) following a recent diagnosis of BPI or SCZ. Findings indicated a trend of patients who received LAIs having lower risk of experiencing all-cause mortality, bankruptcy, or any DLE in the year following a mental health inpatient or ED visit, although the risk difference was less than 2% and did not reach statistical significance (p < 0.10 after propensity score adjustment). These findings also should be interpreted with caution as there were only two instances of outcomes in the LAI arm, one arrest and one judgement filing.

Despite the inability to draw strong conclusions comparing outcomes of LAIs and oral antipsychotics, this study has some important learnings. To our knowledge, this is the first paper assessing DLE outcomes of treated patients with serious mental illness. Prior research has shown that patients with SCZ and BPI have higher rates of DLEs compared to patients with major depressive disorder or the general population [6]. From a clinical perspective, the results of this study indicate that treating patients with SCZ and BPI with LAIs may be beneficial. Prior studies have shown that antipsychotics reduce all-cause mortality in patients with schizophrenia [32,33] and that LAIs are more effective than oral antipsychotics in this regard [34–36]. Our findings seem to support these prior studies. Future confirmative research is warranted.

The population of our study was also young, with a mean age of 22 years old. Research has shown that treating patients with SCZ and BPI early in the disease course is critically important [37]. Delay to first time treatment has been associated with higher levels of depression, and higher number of episodes [38]. The low number clinical outcomes of interest in this study may be due to the fact that our population was young and was treated early in the disease course, preventing further decline. It is also possible that the DLEs of interest were not likely to occur in a population of this age. Future studies of a more diverse age range are warranted.

The primary limitation to the internal validity of this study is its observational design. Without randomized treatment allocation, patients who received an LAI may have differed systematically from those who received oral antipsychotics only. Propensity-score matching improved balance only in the measured characteristics included in the model and could not address unmeasured confounding. In particular, limited or unavailable information on illness severity, socioeconomic status, prior medication adherence, and certain clinical comorbidities may have resulted in residual confounding. Second, the small LAI sample and sparse outcomes substantially reduced statistical power and precision, limited the inferential methods that could be reliably applied, and precluded sensitivity analyses using more nuanced exposure categories. Third, LAI exposure was defined without accounting for formulation, timing, duration, or continuity, and patients classified as most LAI recipients also received oral antipsychotics. The observed associations therefore cannot be solely attributed to LAI therapy. Fourth, the validity and completeness of the DLE were subject to limitations in the underlying public records and their aggregation by third-party vendors. Data availability may have varied across sources and over time, and the absence of a recorded event did not necessarily indicate that the event had not occurred. Although linking public legal and financial records with health records may raise privacy concerns, this study used only publicly available information and followed established procedures to protect patient confidentiality and ensure the ethical use of the linked data. Fifth, the one-year follow-up did not permit evaluation of the longer-term benefits or potential harms of LAI or oral antipsychotic treatment. Finally, this retrospective study did not include patient-reported experiences, treatment preferences, or perceptions of LAI treatment and therefore could not provide a comprehensive assessment of its potential benefits and burdens from the patient perspective.

Due to these limitations, particularly the potential for residual confounding, our findings cannot be interpreted as establishing any causal pathway. Although improved continuity of treatment and medication adherence may represent one possible pathway linking LAI use to DLEs, adherence and the intermediate clinical and social mechanisms underlying this association were not directly evaluated. Future study needs to elucidate the causal pathways that may be driving our results.

There were also several limitations that may restrict external generalizability. The patient population was relatively young and was drawn exclusively from a large integrated health system in Southern California. Treatment patterns and care coordination of patients may be different in an integrated setting than in other healthcare settings. There was also a very low proportion of patients covered by Medicaid in the study sample, and therefore may not be representative of the overall populations of patients with the SCZ or BPI conditions.

## Conclusion

In this exploratory study, receipt of an LAI was associated with lower observed rates of some DLE and mortality outcomes compared with receiving only oral antipsychotic medication without an LAI. However, the small LAI group, sparse outcome events, wide confidence intervals, and potential residual confounding substantially limit the interpretation of these findings. The results should be considered hypothesis-generating and require confirmation in larger studies.
