# Supplementary material for: Long-acting injectable antipsychotics linked to reduced short-term disruptive life event and death
Source: PLOS Ment Health. 2026 Nov 10;3(11):e0000731. doi: 10.1371/journal.pmen.0000731 (PMC13750607; doi:10.1371/journal.pmen.0000731)
Supplement: Supplementary file 1 [file pmen.0000731.s001.docx]

We sincerely thank the reviewers for their constructive and insightful comments. We also need to apologize for our limitations and inability to address all critical and in-depth comments. We have carefully prepared point-by-point responses and revised the manuscript accordingly. The comments are in bold and our responses are in normal font below. We have also highlighted places with major changes in the manuscript.

**Reviewer #1: Review Comments to the Author
This manuscript addresses an important and underexplored topic by examining the association between long-acting injectable antipsychotics (LAIs) and disruptive life events (DLEs) as well as mortality among patients with schizophrenia (SCZ) and bipolar I disorder (BPI). The linkage of electronic health records with external socioeconomic data (e.g., TransUnion records) is innovative and contributes to the growing literature on real-world outcomes in mental health. However, several substantial concerns need to be addressed to improve the scientific rigor, clarity, and interpretability of the findings.**
Thanks for the precise overall summary of our work.

**First, while the study objective is clearly stated, the conceptual framing requires strengthening. The manuscript does not sufficiently justify the theoretical or clinical pathways linking LAIs to disruptive life events such as arrests, bankruptcy, and judgment filings. These outcomes are complex and influenced by multiple social, legal, and economic determinants beyond medication adherence. The authors should elaborate a clearer conceptual model explaining how LAIs might plausibly influence such distal outcomes.**
We thank the reviewer for this important comment. We envisioned and conducted this study an empirical and exploratory investigation based on observational data. A formal and rigorous conceptual model linking antipsychotic medication (including LAI) use to disruptive life events is very important but beyond our knowledge and abilities. Nevertheless, we fully agree that a plausible pathway underlying this association should be presented more clearly in our paper. The primary mechanism hypothesized in the existing literature is that LAIs may improve continuity of antipsychotic medication, which may in turn reduce relapse and psychiatric destabilization. Improvements in mental health stability may potentially reduce the risk of downstream disruptive life events. However, arrests, bankruptcy, and judgment filings are distal and complex outcomes that are also shaped by numerous social, legal, and economic factors. Our study did not directly measure or test any of these pathways. We have therefore added a cautious description of this potential mechanism to the Introduction and clarified in the Discussion that the mechanisms underlying the observed associations remain uncertain.

**Second, the methodological limitations are significant and require more transparent acknowledgment and handling. The very small sample size in the LAI arm (n=218) and extremely low number of outcome events (only two DLE events in the LAI group) severely limit statistical power and the reliability of the findings. Although the authors acknowledge this, the implications are not fully reflected in the interpretation. The use of p<0.10 as a threshold for statistical significance is also questionable and should be more strongly justified or avoided, as it increases the risk of Type I error.**
We fully agree that the small size of the LAI arm and the extremely low number of outcome events substantially limited statistical power and precision of our estimates. We have revised the Abstract, Results, Discussion, and Conclusion to further emphasize these limitations and the exploratory nature of the findings. In particular, we now emphasize the point estimates and 95% confidence intervals (which was reported in Tables 2 and 3 originally) and avoid drawing definitive conclusions from the observed differences. We also agree that p<0.10 is not the conventional threshold for statistical significance. Our original intent was to use this threshold only to identify potentially informative signals in an exploratory study with sparse outcomes, rather than to make confirmatory statistical conclusions. We have clarified this distinction in the Statistical Analysis section. Weak results with p<0.10 have been described as imprecise estimates that may be useful for generating hypotheses for future studies (Results section).

**Third, there are concerns regarding potential selection bias and confounding. The observational design and non-random allocation to LAI versus oral treatment introduce strong risks of confounding by indication. Although propensity score matching was used, the matching variables appear limited (e.g., no inclusion of illness severity, comorbidities, socioeconomic status, or prior adherence behavior). This raises concerns about residual confounding that may bias the results.**
We agree that the observational design can create the potential for selection bias and confounding by indication. Although propensity-score matching improved comparability between the study arms, the improved balance is limited to measured covariates only, and residual confounding due to unobserved characteristics can still exist. In particular, information on illness severity, socioeconomic status, prior medication adherence, and clinical comorbidities was either unavailable (e.g., illness severity) or not usable (e.g., too few non-commercially insured) to us. Residual confounding may therefore remain and may have influenced the estimated associations. We have expanded the Discussion to acknowledge this limitation more explicitly and have revised the interpretation of the findings to avoid implying that propensity-score matching established causal comparability between the treatment groups.

**Fourth, the definition of exposure (LAI use within 180 days post-index event) may introduce misclassification bias. Patients in the LAI arm also received oral antipsychotics, which complicates attribution of effects solely to LAIs. A more nuanced exposure definition or sensitivity analyses (e.g., timing, duration, or exclusive LAI use) would strengthen the analysis.**
We agree that defining LAI exposure as receipt of an LAI within 180 days after the index event does not capture variation in the timing, duration, or continuity of LAI treatment. We also agree that patients in the LAI group could receive oral antipsychotics concurrently. Therefore, the exposure contrast in this study should be interpreted as receipt of an LAI, with or without concurrent oral antipsychotic treatment, versus receipt of oral antipsychotic treatment without an LAI, rather than as exclusive LAI treatment versus exclusive oral treatment. LAI exposure was defined based on any LAI receipt within 180 days after the index event, without consideration of the timing, duration, or continuity of LAI treatment. We have further clarified this exposure definition throughout the manuscript and revised the interpretation to avoid attributing the observed associations solely to LAI therapy.

More nuanced exposure classifications and sensitivity analyses based on the timing, duration, or exclusive use of LAIs would be informative. We do have the pharmacy dispensation data for the detailed classification. However, we do not have sufficient sample size to support any statistical analysis. As you previously commented, the current and coarse LAI arm definition already had limited statistical power and very restricted statistical inference. We therefore were unable to pursue more detailed exposure definition in this study. We have acknowledged this limitation in the Discussion and identified it as an important area for investigation in larger studies.

**Fifth, the outcome measures—particularly DLEs derived from credit and legal records—require further clarification. The validity, completeness, and potential biases in linking TransUnion data to clinical records should be discussed in greater detail. Additionally, the ethical implications of using such data sources should be explicitly addressed.**

The TransUnion data source and linkage procedures was developed and evaluated in our prior publication, which described these issues in detail (online supplement of Nau et al., 2023, JAMA Psychiatry, reference 6 in this paper). The DLE data were derived from publicly available court and law-enforcement records aggregated by TransUnion and did not include credit reports, credit scores, or other nonpublic financial information. More than 98% of eligible patient records were linked to the TransUnion data. A multistep data-transfer process was used to separate identifying information from clinical information and to create a de-identified analytic data set). The present study reused the previously established DLE outcome data. We have revised the Methods to summarize these procedures and direct readers to the prior publication and its supplement for full details. We have also expanded the Discussion to acknowledge that the completeness of public-record data may vary across sources and over time and that events absent from these records cannot necessarily be assumed not to have occurred. Finally, we have added a discussion of the ethical considerations associated with linking health records to legal and financial public-record data, including patient privacy, confidentiality, and preferences concerning the use of such information.

**Sixth, the generalizability of the findings is limited. The study population is relatively young (mean age ~22 years), drawn from a single integrated healthcare system, and has low Medicaid representation. These factors should be more critically discussed in relation to broader populations with SCZ and BPI.**
We expanded the limitation discussion to further emphasize these limitations (last paragraph in Discussion, highlighted).

**Seventh, the interpretation of results tends to overstate the findings. The conclusions suggest that LAIs “may improve” outcomes, yet the results are largely non-significant and based on extremely sparse data. The language should be revised to reflect the exploratory nature of the study and the high level of uncertainty.**
We apologize for the overstatement in the original Conclusion section. Given all limitations, the findings cannot provide sufficient evidence that LAI treatment improves DLEs or mortality. We have revised the Abstract, Discussion, and Conclusion to remove overreaching statements suggesting that LAIs may improve these outcomes. The revised manuscript characterizes the results as exploratory associations with substantial uncertainty and emphasizes that they should be interpreted as hypothesis-generating rather than significant evidence.

**Finally, issues related to research and publication ethics should be clarified. Although funding sources are disclosed, the involvement of funders in study design and manuscript preparation raises potential concerns about bias and should be discussed more transparently. The data availability statement also does not fully align with PLOS policies, as the data are not publicly accessible; the authors should provide a clearer justification and indicate whether data access can be granted under reasonable conditions.**
We have revised the Funding Statement to specify the funders’ roles in the study design and manuscript development. Although representatives of the funders participated in discussions regarding the study design, interpretation of the results, and review of the manuscript, the scientific investigator team retained responsibility for the analysis, interpretation of the findings, decision to submit the manuscript, and final content of the manuscript.

We have also revised the Data Availability Statement. The individual-level data cannot be made publicly available because they contain protected health information and were obtained under institutional and third-party data-use agreements that do not permit public disclosure. Qualified researchers may request access through the applicable institutional review and data-access processes, subject to approval and execution of the necessary data-use agreements. The revised statement provides the appropriate contact and access information.

**Summary Recommendation
The manuscript addresses a novel and relevant research question with an innovative dataset. However, major revisions are required to address concerns related to conceptual framing, methodological rigor, statistical validity, interpretation of findings, and transparency. Strengthening these areas will significantly improve the manuscript’s contribution to the field.**

We thank you again for the constructive and comprehensive comments. We have made revisions throughout the paper to address your suggestions. We believe that this paper has been substantially improved by incorporating your suggestions.

**Reviewer #2: Thank you for the opportunity to review this paper. I would like to acknowledge the effort and commitment of the authors in addressing an important and complex topic. While the study presents interesting findings, I have several concerns that I believe should be addressed to strengthen the work.**
Many thanks for your encouragement. Please check our detailed response below.

**My primary concern relates not only to what is included in the study, but also to what appears to be excluded. First, LAI antipsychotics are not clearly defined in terms of duration of action, administration protocols, or care processes. Greater clarity is needed regarding how long these injections last, how frequently they require clinical oversight, and where and how they are typically administered. Given that the study focuses on short-term outcomes, it would also be important to clearly outline, if not all, but some of the short-term side effects associated with LAIs.**
We thank the reviewer for raising these important clinical considerations. Since this and a few later comments share the same concern, we feel it is important to clarify the scope and purpose of the present study. The present study was an empirical, retrospective, observational analysis of existing EHR and public-record data designed to examine associations between documented LAI receipt and subsequent DLEs and mortality. It was not designed to evaluate individual LAI formulations, duration of pharmacologic action, administration protocols, clinical oversight, care settings, or clinical adverse effects. These characteristics vary across LAI medications and were not available in essential detail in our research data to support reliable comparative analyses.

We have revised the Methods to clarify our operational definition of LAI exposure and the scope of the study. We have also added to the Discussion that differences in LAI formulation, administration frequency, care processes, and adverse effects were not evaluated and may affect treatment experiences and outcomes. A comprehensive examination of these clinical dimensions is beyond the scope of the present empirical study and will require studies specifically designed to evaluate them.

**Relatedly, the method of administration is a significant omission. In many cases, particularly in the context of psychosis, injectable antipsychotics may be administered involuntarily. If this occurred within the study population, factors such as coercion or hospitalization could themselves influence disruptive life events (DLEs), yet these variables are not accounted for in the analysis. Although the current research landscape offers limited means of systematically capturing these dynamics, their omission should be explicitly acknowledged, as they may meaningfully shape the outcomes reported.**
We thank the reviewer for raising this very important clinical issue. To our knowledge, involuntary administration of an LAI during a mental health emergency is generally limited to uncommon circumstances in which short-acting oral or injectable medications are not clinically appropriate. However, the secondary EHR data did not allow us to assess whether an LAI or oral medication was administered voluntarily or involuntarily. We could not evaluate coercion or involuntary treatment as a separate exposure or potential confounding factor.

**More broadly, the study does not appear to incorporate patient perspectives, which represents a notable limitation. By focusing primarily on clinical outcomes and narrowly defined DLEs, the research may overlook how patients themselves experience and interpret the impact of LAI treatment. Given the study design, patient perspectives may not be feasible to include; however, this should also be more explicitly acknowledged as a limitation, as their absence restricts a more balanced understanding of the findings.**

We agree that patient perspectives are important for understanding how patients experience LAI treatment and evaluate its effects on their lives. However, the present study was a retrospective analysis of existing EHR and public-record data and therefore did not include patient-reported experiences, treatment preferences, or perceptions of LAI treatment. We have added this issue to the limitations and clarified that the selected outcomes do not provide a comprehensive assessment of treatment benefits and burdens from the patient’s perspective. Future studies incorporating patient-reported outcomes and qualitative data would provide a more balanced understanding of the effects of LAI treatment.

**Although the study is concerned with short-term outcomes, some contextual reference to the longer-term effects of antipsychotic treatment would be valuable. There is an increasing emphasis in the literature on the potential harms associated with psychotropic medications, and acknowledging this would help situate the findings more comprehensively and with greater balance.**
Thank you for highlighting this important direction for future research. Our study focused on DLEs and mortality during the 12 months after the index event and was not designed to evaluate the longer-term benefits or potential harms of antipsychotic treatment. We agree that the short follow-up period provides an incomplete assessment of the overall balance of treatment benefits and harms. We have added this limitation to the Discussion and clarified that our findings should not be extrapolated to the longer-term effects of LAI or oral antipsychotic treatment. Future studies with longer follow-up are needed to evaluate these effects more comprehensively.

**I am also concerned about potential misrepresentation in the introduction. The statement that “many patients and mental health care providers were found to have somewhat unfavorable perceptions of LAIs” appears to rely on a study that included a limited sample, including 25 psychiatrists. While the findings may reflect the perspectives within that study, the phrasing risks overstating or generalizing these views. Greater precision in how this evidence is presented would improve accuracy. Additionally, the same referenced study discusses the “coercive image” of LAIs, which further underscores the importance of addressing how these medications are administered. An issue not sufficiently explored in the current paper.**
We agree that the original wording in Introduction generalized beyond the population included in the cited small study. We have revised the Introduction to describe the finding as specific to the patients and psychiatrists who participated in that study, rather than as representative of patients and mental health care providers more broadly. We also acknowledge the reviewer’s concern regarding the “coercive image” associated with LAIs. The present study was an empirical analysis of existing EHR and public-record data and could not evaluate the circumstances under which LAIs were administered. In particular, the available data did not capture whether LAI administration was voluntary or involuntary or whether patients perceived the treatment as coercive. We have added this limitation to the Discussion.

**Finally, there are broader ethical considerations worth noting. The use of personal and health records, while valuable for observational research, inherently excludes subjective patient experiences that cannot be captured through quantitative data alone. Furthermore, the classification of DLEs (e.g., arrests, bankruptcy, judgments) raises questions about how these categories were selected and whether they adequately account for broader social and structural determinants of health. Without this context, there is a risk of reinforcing stigma associated with severe mental illness.**
We thank the reviewer for highlighting these broader considerations. The DLE categories examined in this study, including arrests, bankruptcy filings, and judgment filings, were primarily based on the availability of objectively recorded public-record data and were established in our previous work using the TransUnion data source (reference 6 in the paper). They were not intended to characterize patients or to provide a comprehensive measure of their social circumstances or lived experiences.

We agree that these administrative outcomes do not capture patients’ subjective experiences or the broader social and structural factors that may contribute to their occurrence. The present study was an empirical analysis of existing EHR and public-record data and was not designed to evaluate these mechanisms. We have revised the Discussion to clarify that DLEs are complex outcomes shaped by social, economic, legal, and structural determinants beyond antipsychotic treatment. We have also acknowledged that patient perspectives were unavailable and that the findings should not be interpreted as attributing DLEs to individual patients or their mental health conditions.

**In summary, while the study addresses an important area, there is considerable scope for expansion and refinement. Greater clarity, contextualization, and reflexivity, particularly regarding methodology, interpretation, and ethical considerations, would significantly strengthen the paper.
I hope these comments are received in the constructive spirit in which they are intended, and I appreciate the opportunity to engage with this work.**

We sincerely thank the reviewer for these insightful and constructive comments. We fully acknowledge that several important clinical and patient-centered questions could not be addressed within the scope of this retrospective empirical study or using the available data. We have clarified these limitations in the revised manuscript and identified them as important areas for future research.

**Reviewer #3: Great study that will add much to the decision of clinicians whether to use LAI or not.
The structure of the article is logical, with each section building upon the previous one, making it easy for readers to understand the progression of ideas. This is an informative article that I believe it will contribute well to the field.**

Many thanks for your positive feedback. We hope that this paper can contribute useful empirical data to the literature and can aid the decision for clinicians and patients.
