# Supplementary material for: Long-acting injectable antipsychotics linked to reduced short-term disruptive life event and death
Source: PLOS Ment Health. 2026 Nov 10;3(11):e0000731. doi: 10.1371/journal.pmen.0000731 (PMC13750607; doi:10.1371/journal.pmen.0000731)
Supplement: Peer review history

# Peer review: Decision Letter 0

**DOI:** 10.1371/journal.pmen.0000731.r001

**Authors:** Jenna Scaramanga

25 May 2026

PMEN-D-26-00108

Long-Acting Injectable Antipsychotics Linked to Reduced Short-Term Disruptive Life Event and Death

PLOS Mental Health

Dear Dr. Han,

Thank you for submitting your manuscript to PLOS Mental Health. After careful consideration, we feel that it has merit but does not fully meet PLOS Mental Health’s publication criteria as it currently stands. Therefore, we invite you to submit a revised version of the manuscript that addresses the points raised during the review process.

Please carefully revise your manuscript to address the points raised by the reviewers.

Please submit your revised manuscript by Jul 20 2026 11:59PM. If you will need more time than this to complete your revisions, please reply to this message or contact the journal office at mentalhealth@plos.org. When you're ready to submit your revision, log on to https://www.editorialmanager.com/pmen/ and select the 'Submissions Needing Revision' folder to locate your manuscript file.

Please include the following items when submitting your revised manuscript:

- A letter that responds to each point raised by the editor and reviewer(s). You should upload this letter as a separate file labeled 'Response to Reviewers'.
- A marked-up copy of your manuscript that highlights changes made to the original version. You should upload this as a separate file labeled 'Revised Manuscript with Track Changes'.
- An unmarked version of your revised paper without tracked changes. You should upload this as a separate file labeled 'Manuscript'.

Guidelines for resubmitting your figure files are available below the reviewer comments at the end of this letter.

As the corresponding author, your ORCID iD is verified in the submission system and will appear in the published article. PLOS supports the use of ORCID, and we encourage all coauthors to register for an ORCID iD and use it as well. Please encourage your coauthors to verify their ORCID iD within the submission system before final acceptance, as unverified ORCID iDs will not appear in the published article. *Only*  the individual author can complete the verification step; PLOS staff *cannot*  verify ORCID iDs on behalf of authors.

We look forward to receiving your revised manuscript.

Kind regards,

Jenna Scaramanga

Staff Editor

PLOS Mental Health

Journal Requirements:

1. Please provide a complete Data Availability Statement in the submission form, ensuring you include all necessary access information or a reason for why you are unable to make your data freely accessible. If your research concerns only data provided within your submission, please write "All data are in the manuscript and/or supporting information files" as your Data Availability Statement.

2. We have noticed that you have uploaded Supporting Information files, but you have not included a list of legends. Please add a full list of legends for your Supporting Information files after the references list.

3. Please provide a complete Data Availability Statement in the submission form, ensuring you include all necessary access information or a reason for why you are unable to make your data freely accessible. If your research concerns only data provided within your submission, please write "All data are in the manuscript and/or supporting information files" as your Data Availability Statement.

4. Please provide separate figure files in .tif or .eps format.

For more information about figure files please see our guidelines:  LINK

https://journals.plos.org/mentalhealth/s/figures

https://journals.plos.org/mentalhealth/s/figures#loc-file-requirements

If the reviewer comments include a recommendation to cite specific previously published works, please review and evaluate these publications to determine whether they are relevant and should be cited. There is no requirement to cite these works unless the editor has indicated otherwise.

Additional Editor Comments (if provided):

[Note: HTML markup is below. Please do not edit.]

Reviewers' comments:

Reviewer's Responses to Questions

**Comments to the Author**

1. Does this manuscript meet PLOS Mental Health’s publication criteria? Is the manuscript technically sound, and do the data support the conclusions? The manuscript must describe methodologically and ethically rigorous research with conclusions that are appropriately drawn based on the data presented.

Reviewer #1: Yes

Reviewer #2: Partly

Reviewer #3: Yes

**********

2. Has the statistical analysis been performed appropriately and rigorously?

Reviewer #1: Yes

Reviewer #2: Yes

Reviewer #3: Yes

**********

3. Have the authors made all data underlying the findings in their manuscript fully available (please refer to the Data Availability Statement at the start of the manuscript PDF file)?

The PLOS Data policy requires authors to make all data underlying the findings described in their manuscript fully available without restriction, with rare exception. The data should be provided as part of the manuscript or its supporting information, or deposited to a public repository. For example, in addition to summary statistics, the data points behind means, medians and variance measures should be available. If there are restrictions on publicly sharing data—e.g. participant privacy or use of data from a third party—those must be specified.

Reviewer #1: Yes

Reviewer #2: Yes

Reviewer #3: Yes

**********

4. Is the manuscript presented in an intelligible fashion and written in standard English?

PLOS Mental Health does not copyedit accepted manuscripts, so the language in submitted articles must be clear, correct, and unambiguous. Any typographical or grammatical errors should be corrected at revision, so please note any specific errors here.

Reviewer #1: Yes

Reviewer #2: Yes

Reviewer #3: Yes

**********

5. Review Comments to the Author

Please use the space provided to explain your answers to the questions above. You may also include additional comments for the author, including concerns about dual publication, research ethics, or publication ethics. (Please upload your review as an attachment if it exceeds 20,000 characters)

Reviewer #1: Review Comments to the Author

This manuscript addresses an important and underexplored topic by examining the association between long-acting injectable antipsychotics (LAIs) and disruptive life events (DLEs) as well as mortality among patients with schizophrenia (SCZ) and bipolar I disorder (BPI). The linkage of electronic health records with external socioeconomic data (e.g., TransUnion records) is innovative and contributes to the growing literature on real-world outcomes in mental health. However, several substantial concerns need to be addressed to improve the scientific rigor, clarity, and interpretability of the findings.

First, while the study objective is clearly stated, the conceptual framing requires strengthening. The manuscript does not sufficiently justify the theoretical or clinical pathways linking LAIs to disruptive life events such as arrests, bankruptcy, and judgment filings. These outcomes are complex and influenced by multiple social, legal, and economic determinants beyond medication adherence. The authors should elaborate a clearer conceptual model explaining how LAIs might plausibly influence such distal outcomes.

Second, the methodological limitations are significant and require more transparent acknowledgment and handling. The very small sample size in the LAI arm (n=218) and extremely low number of outcome events (only two DLE events in the LAI group) severely limit statistical power and the reliability of the findings. Although the authors acknowledge this, the implications are not fully reflected in the interpretation. The use of p<0.10 as a threshold for statistical significance is also questionable and should be more strongly justified or avoided, as it increases the risk of Type I error.

Third, there are concerns regarding potential selection bias and confounding. The observational design and non-random allocation to LAI versus oral treatment introduce strong risks of confounding by indication. Although propensity score matching was used, the matching variables appear limited (e.g., no inclusion of illness severity, comorbidities, socioeconomic status, or prior adherence behavior). This raises concerns about residual confounding that may bias the results.

Fourth, the definition of exposure (LAI use within 180 days post-index event) may introduce misclassification bias. Patients in the LAI arm also received oral antipsychotics, which complicates attribution of effects solely to LAIs. A more nuanced exposure definition or sensitivity analyses (e.g., timing, duration, or exclusive LAI use) would strengthen the analysis.

Fifth, the outcome measures—particularly DLEs derived from credit and legal records—require further clarification. The validity, completeness, and potential biases in linking TransUnion data to clinical records should be discussed in greater detail. Additionally, the ethical implications of using such data sources should be explicitly addressed.

Sixth, the generalizability of the findings is limited. The study population is relatively young (mean age ~22 years), drawn from a single integrated healthcare system, and has low Medicaid representation. These factors should be more critically discussed in relation to broader populations with SCZ and BPI.

Seventh, the interpretation of results tends to overstate the findings. The conclusions suggest that LAIs “may improve” outcomes, yet the results are largely non-significant and based on extremely sparse data. The language should be revised to reflect the exploratory nature of the study and the high level of uncertainty.

Finally, issues related to research and publication ethics should be clarified. Although funding sources are disclosed, the involvement of funders in study design and manuscript preparation raises potential concerns about bias and should be discussed more transparently. The data availability statement also does not fully align with PLOS policies, as the data are not publicly accessible; the authors should provide a clearer justification and indicate whether data access can be granted under reasonable conditions.

Summary Recommendation

The manuscript addresses a novel and relevant research question with an innovative dataset. However, major revisions are required to address concerns related to conceptual framing, methodological rigor, statistical validity, interpretation of findings, and transparency. Strengthening these areas will significantly improve the manuscript’s contribution to the field.

Reviewer #2: Thank you for the opportunity to review this paper. I would like to acknowledge the effort and commitment of the authors in addressing an important and complex topic. While the study presents interesting findings, I have several concerns that I believe should be addressed to strengthen the work.

My primary concern relates not only to what is included in the study, but also to what appears to be excluded. First, LAI antipsychotics are not clearly defined in terms of duration of action, administration protocols, or care processes. Greater clarity is needed regarding how long these injections last, how frequently they require clinical oversight, and where and how they are typically administered. Given that the study focuses on short-term outcomes, it would also be important to clearly outline, if not all, but some of the short-term side effects associated with LAIs.

Relatedly, the method of administration is a significant omission. In many cases, particularly in the context of psychosis, injectable antipsychotics may be administered involuntarily. If this occurred within the study population, factors such as coercion or hospitalization could themselves influence disruptive life events (DLEs), yet these variables are not accounted for in the analysis. Although the current research landscape offers limited means of systematically capturing these dynamics, their omission should be explicitly acknowledged, as they may meaningfully shape the outcomes reported.

More broadly, the study does not appear to incorporate patient perspectives, which represents a notable limitation. By focusing primarily on clinical outcomes and narrowly defined DLEs, the research may overlook how patients themselves experience and interpret the impact of LAI treatment. Given the study design, patient perspectives may not be feasible to include; however, this should also be more explicitly acknowledged as a limitation, as their absence restricts a more balanced understanding of the findings.

Although the study is concerned with short-term outcomes, some contextual reference to the longer-term effects of antipsychotic treatment would be valuable. There is an increasing emphasis in the literature on the potential harms associated with psychotropic medications, and acknowledging this would help situate the findings more comprehensively and with greater balance.

I am also concerned about potential misrepresentation in the introduction. The statement that “many patients and mental health care providers were found to have somewhat unfavorable perceptions of LAIs” appears to rely on a study that included a limited sample, including 25 psychiatrists. While the findings may reflect the perspectives within that study, the phrasing risks overstating or generalizing these views. Greater precision in how this evidence is presented would improve accuracy. Additionally, the same referenced study discusses the “coercive image” of LAIs, which further underscores the importance of addressing how these medications are administered. An issue not sufficiently explored in the current paper.

Finally, there are broader ethical considerations worth noting. The use of personal and health records, while valuable for observational research, inherently excludes subjective patient experiences that cannot be captured through quantitative data alone. Furthermore, the classification of DLEs (e.g., arrests, bankruptcy, judgments) raises questions about how these categories were selected and whether they adequately account for broader social and structural determinants of health. Without this context, there is a risk of reinforcing stigma associated with severe mental illness.

In summary, while the study addresses an important area, there is considerable scope for expansion and refinement. Greater clarity, contextualization, and reflexivity, particularly regarding methodology, interpretation, and ethical considerations, would significantly strengthen the paper.

I hope these comments are received in the constructive spirit in which they are intended, and I appreciate the opportunity to engage with this work.

Reviewer #3: Great study that will add much to the decision of clinicians whether to use LAI or not.

The structure of the article is logical, with each section building upon the previous one, making it easy for readers to understand the progression of ideas. This is an informative article that I believe it will contribute well to the field.

**********

6. PLOS authors have the option to publish the peer review history of their article (what does this mean?). If published, this will include your full peer review and any attached files.

**Do you want your identity to be public for this peer review?**  If you choose “no”, your identity will remain anonymous but your review may still be made public.

For information about this choice, including consent withdrawal, please see our Privacy Policy.

Reviewer #1: No

Reviewer #2: No

Reviewer #3: No

**********

[NOTE: If reviewer comments were submitted as an attachment file, they will be attached to this email and accessible via the submission site. Please log into your account, locate the manuscript record, and check for the action link "View Attachments". If this link does not appear, there are no attachment files.]

-->Figure Resubmissions:

While revising your submission, we strongly recommend that you use PLOS’s NAAS tool (https://ngplosjournals.pagemajik.ai/artanalysis) to test your figure files. NAAS can convert your figure files to the TIFF file type and meet basic requirements (such as print size, resolution), or provide you with a report on issues that do not meet our requirements and that NAAS cannot fix.

After uploading your figures to PLOS’s NAAS tool - https://ngplosjournals.pagemajik.ai/artanalysis, NAAS will process the files provided and display the results in the "Uploaded Files" section of the page as the processing is complete. If the uploaded figures meet our requirements (or NAAS is able to fix the files to meet our requirements), the figure will be marked as "fixed" above. If NAAS is unable to fix the files, a red "failed" label will appear above. When NAAS has confirmed that the figure files meet our requirements, please download the file via the download option, and include these NAAS processed figure files when submitting your revised manuscript.

# Author comment: Author response to Decision Letter 1

**DOI:** 10.1371/journal.pmen.0000731.r002

10 Aug 2026

Attachment Submitted filename: point by point response.docx

# Editor report: Decision Letter 1

**DOI:** 10.1371/journal.pmen.0000731.r003

**Authors:** Jenna Scaramanga

2 Sep 2026

Long-Acting Injectable Antipsychotics Linked to Reduced Short-Term Disruptive Life Event and Death

PMEN-D-26-00108R1

Dear Dr. Han,

We are pleased to inform you that your manuscript 'Long-Acting Injectable Antipsychotics Linked to Reduced Short-Term Disruptive Life Event and Death' has been provisionally accepted for publication in PLOS Mental Health.

Before your manuscript can be formally accepted you will need to complete some formatting changes, which you will receive in a follow up email. A member of our team will be in touch with a set of requests.

Please note that your manuscript will not be scheduled for publication until you have made the required changes, so a swift response is appreciated.

IMPORTANT: The editorial review process is now complete. PLOS will only permit corrections to spelling, formatting or significant scientific errors from this point onwards. Requests for major changes, or any which affect the scientific understanding of your work, will cause delays to the publication date of your manuscript.

If your institution or institutions have a press office, please notify them about your upcoming paper to help maximize its impact. If they'll be preparing press materials, please inform our press team as soon as possible -- no later than 48 hours after receiving the formal acceptance. Your manuscript will remain under strict press embargo until 2 pm Eastern Time on the date of publication. For more information, please contact mentalhealth@plos.org.

Thank you again for supporting Open Access publishing; we are looking forward to publishing your work in PLOS Mental Health.

Best regards,

Karli Montague-Cardoso

Staff Editor

PLOS Mental Health

***********************************************************

Reviewer Comments (if any, and for reference):
